# DAILY SLEEP AND EMOTION INERTIA IN LATE LIFE

**DOI:** 10.1093/geroni/igae098.3518

**Published:** 2024-12-31

**Authors:** Zexi Zhou, Karen Fingerman

**Affiliations:** The University of Texas Austin, Austin, Texas, United States; The University of Texas Austin, Austin, Texas, United States

## Abstract

Emotion inertia, conceptualized as the extent to which specific emotional states persist over time, is a crucial aspect of emotion dynamics associated with psychological well-being. This study examines the associations between sleep and emotion inertia in older adults’ daily life. We used the ecological momentary assessment data over 5–6 days from the Daily Experiences and Well-being Study (N = 251, Mage = 73.7). Each morning, older adults indicated their sleep (i.e., duration, quality, disturbances) the prior night. At each three-hour assessment, they rated feelings of positive (e.g., loved, content) and negative (e.g., worried, lonely) emotion. They also reported depressive symptoms in the baseline interview. Multilevel models showed that higher sleep quality was associated with lower emotion inertia on days when older adults had lower averaged positive emotion or higher averaged negative emotion. In contrast, on days featuring higher averaged positive emotion or lower averaged negative emotion, high sleep quality was associated with increased emotion inertia. Furthermore, more overall hours of sleep predicted less emotion inertia across the study period only among older adults who had more depressive symptoms but not among those with fewer depressive symptoms. Findings suggest that good sleep may benefit older adults’ everyday emotional well-being by maintaining good mood on days characterized by favorable emotional states, and facilitating susceptibility to change in mood on days with less favorable emotional states. Results also underscore the heightened protective role of good sleep in older adults experiencing more depressive symptoms.

